# COVID-19 and Italian Healthcare Workers From the Initial Sacrifice to the mRNA Vaccine: Pandemic Chrono-History, Epidemiological Data, Ethical Dilemmas, and Future Challenges

**DOI:** 10.3389/fpubh.2020.591900

**Published:** 2021-01-21

**Authors:** Matteo Nioi, Pietro Emanuele Napoli, Jessica Lobina, Maurizio Fossarello, Ernesto d'Aloja

**Affiliations:** ^1^Department of Medical Sciences and Public Health, Forensic Medicine Unit, University of Cagliari, Cagliari, Italy; ^2^Department of Surgical Science, Eye Clinic, University of Cagliari, Cagliari, Italy

**Keywords:** COVID-19, COVID-19 healthcare workers, COVID-19: specialties of dead doctors, COVID-19 future challenges, COVID-19 mRNA vaccine, COVID-19 Italian physician's positivities and deaths, COVID-19 ethical dilemmas, COVID-19 HCWs deaths

## Abstract

On March 11, 2020, the World Health Organization (WHO) declared the coronavirus disease 2019 (COVID-19) outbreak a pandemic. Simultaneously, in Italy, in which the first case had occurred on February 18, the rigid phase of the lockdown began. The country has attracted worldwide attention, becoming at the same time a field of study both concerning the spread of the pandemic and advanced assessments of the effectiveness of political, public health, and therapeutic measures. The protagonists of the Italian crisis were the healthcare workers (HCWs) who were exposed to severe acute respiratory syndrome coronavirus 2 (SARS-CoV-2) without having any perception of what they were facing, courageously contributing to the containment of the epidemic to be defined by the media as “heroes.” However, in the first phase of the pandemic (March–May 2020), the price that the Italian Public Health System had to pay both in terms of the number of positive virus cases and deaths among the HCWs was beyond and represented a peculiarity compared to what happened in other countries. In the current study, after a summary of the evolution of the pandemic in Italy, we offer an analysis of the statistical data concerning contagions and deaths among healthcare workers (physicians in particular). In conclusion, we describe the critical issues that still need to be resolved and the future challenges facing healthcare workers and the general population.

## Introduction: Epidemic Chrono-History and the Evolution of the Italian Scenario

The first domestic case of COVID-19 was detected on February 21 in a 38-year-old man from Lombardy (1). Thereafter, the local epidemic expanded rapidly to the neighboring areas with an estimated basic reproduction number (R0) of between 2.43 and 3.10 (2). A difference in terms of incidence began to emerge between the Northern and the Southern regions of Italy. Different hypotheses have been proposed to explain this inhomogeneous distribution of cases from a demographic, geographic, and genetic perspective (3–5). Although the Italian Government-mandated containment restriction extended to all national territories on March 11, on March 19, Italy overtook China in the number of deaths due to coronavirus disease 2019 (COVID-19) (3,405) and was (temporarily) the country with the most deaths due to the disease.

Early epidemic phases in Italy were characterized by widespread unpreparedness of the National Healthcare System (NHS) for a similar large-scale event [such as ICU beds, personal protective equipment, and healthcare workers (HCW)]. These NHS shortcomings led HCWs to apply a very selective triage procedure to patients requiring invasive respiratory support to decide who to “treat” with the best available means and who to “palliate” based on the highest probability to survive. In the attempt to unburden attending physicians of the weight of their ethical and deontological decisions, the more prominent Italian Scientific Society in the Intensive Care context (SIAARTI) drew up a recommendation addressing the fair allocation of scarce medical resources (6, 7).

Likely, a profound and irreparable health crisis was avoided by the lockdown, the advent of new therapies, and the widespread distribution of PPE among staff. In contrast, later phases were initiated with a progressive increase in daily recovering people and appeared in conjunction with better knowledge about viral features and an increase in the availability of medical resources.

**Figure 1:**
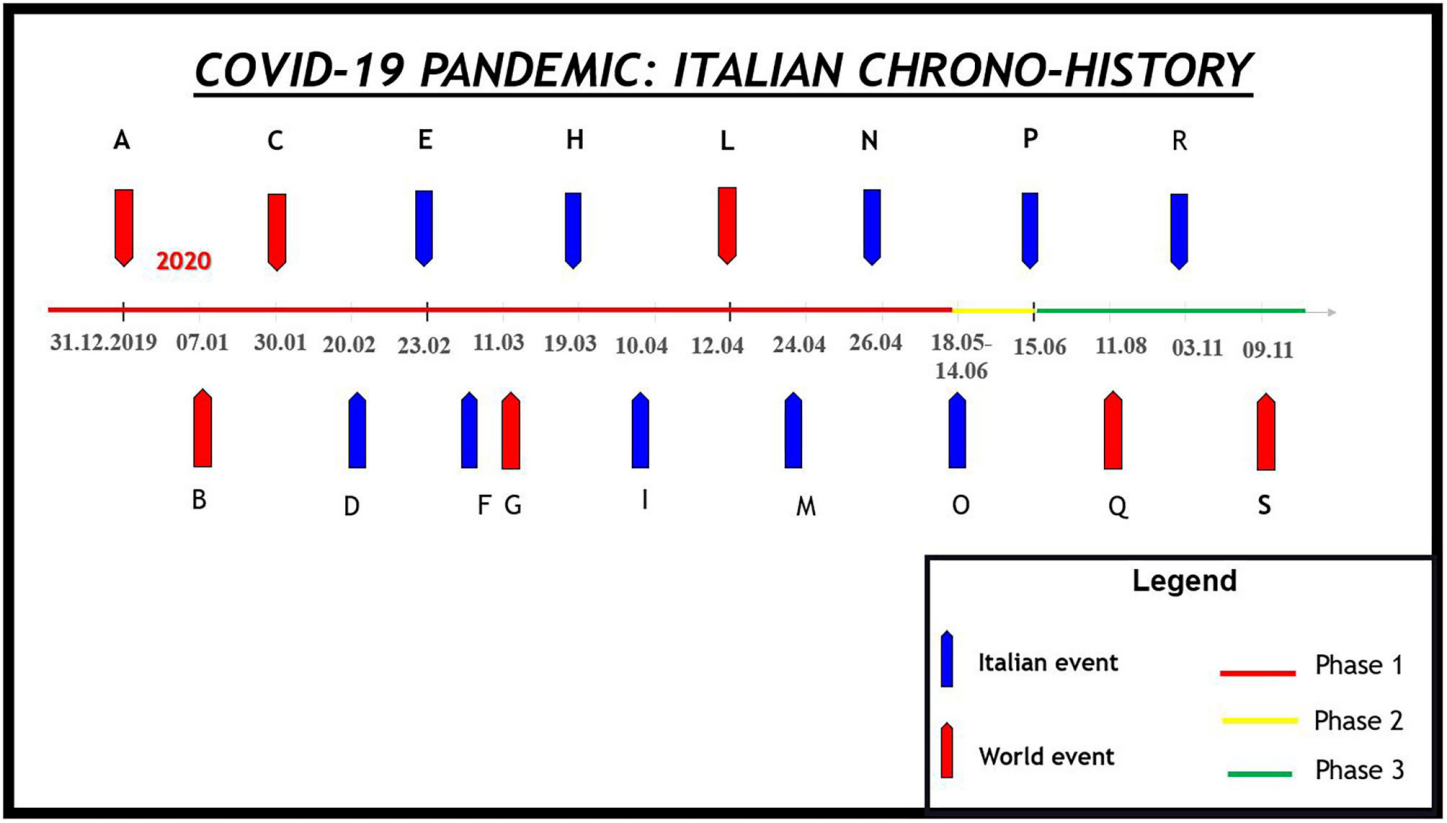
The coronavirus disease 2019 (COVID-19) pandemic Italian chrono-history. **(A)** The Chinese Health authority informs the WHO about 41 patients with mysterious pneumonia. **(B)** Chinese authorities identify a new type of coronavirus. **(C)** The WHO declares a global public health emergency. **(D)** The first case of COVID-19 in Italy. **(E)** The Italian government extends restrictions on mobility and assembly of the person in restricted regions of the country. **(F)** The WHO declares COVID-19 a pandemic. **(G)** The Italian government extends restrictions on mobility and assembly of persons across the whole nation: the Italian lockdown began. **(H)** Italy becomes the world leader in COVID-19 deaths. **(I)** The Italian lockdown is extended until May 4, 2020. **(L)** For the first time in history, Easter Mass is celebrated without worshipers to prevent contagion. The mass was shared on various communications routes (to avoid what happened in the Black Plague). **(M)** For the first time, the number of recovered patients was higher than the number of new cases. **(N)** The Italian government announces the end of the first phase of the pandemic and the beginning of a second deal from May 4, 2020. **(O)** Italian Phase II. Easing of restrictive measures. **(P)** Italian Phase III. Period of coexistence with the SARS-CoV2. **(Q)** Russia became the first country to approve a vaccine against severe acute respiratory syndrome coronavirus 2. **(R)** Announcement of the effectiveness of a vaccine produced by an American pharmaceutical company. **(S)** New restrictive measures (including national night curfew) differentiated according to the situation in the different regions.

The progressive containment of the pandemic has been achieved through the establishment of a rigid lockdown (March 9–May 3, 2020; Italian Phase I) followed by a phase of mitigation of the measures (May 4–June 14; Italian Phase II), and finally, from 15 June, the phase of coexistence with the severe acute respiratory syndrome coronavirus 2 (SARS-CoV-2). However, the increase in the number of cases led to new restrictive measures in November 2020 (Figure 1).

Pandemic and restrictive measures have led to important economic and social changes in the country (8–10).

The report released on 25 November 2020 describes 1,454,529 confirmed cases, 49,931 deaths, and 66,618 cases for COVID-19 in healthcare workers (HCWs) (Istituto Superiore di Sanita Epidemia COVID-19).

## Epidemiological Data: the Sacrifice of Italian Family Doctors

### Description of the Data Source

An important factor to consider for understanding the impact of COVID-19 on the health system is the percentage of COVID-infected HCWs. The dimension of the phenomenon regarding the number of affected and deceased health professionals can be obtained by consulting various data sources. The general data concerning the Italian population's data in general as regards the number of positive individuals, the number of deaths, and the number of positive health workers were obtained through the data provided by the “Istituto Superiore di Sanità” (ISS) (11). The data concerning the work subcategories were obtained by analyzing the data of the “National Institute for Accident Insurance” (INAIL), the Italian Insurance Institute that awards workers in the event of accidents and occupational diseases. As far as health is concerned, employee workers in public or private structures are protected by the Institute (12). Unfortunately, some figures relevant to public health, such as general practitioners, are not covered by the institution.

The data concerning the deaths of doctors are instead obtained from the archive of the “Federazione Nazionale degli Ordini dei Medici Chirurghi e degli Odontoiatri” (FNOMCeO). This archive appears to be the most complete and reliable because anyone practicing the profession in Italy must register. As of January 1st, 2020, there were 403,454 members. During the pandemic, the FNEOMCEO reported the name of every Italian doctor that has died, whether employed or freelance, and the data related to the type of specialization (13).

### COVID-19 and HCWs in Italy: The Report by INAIL

Data from ISS daily reports say HCWs made up 12% of positive patients in July (14). The percentage value will change if we consider the post-lockdown period (June–September period), during which healthcare professionals made up 4.5% of positive patients nationwide (11). The INAIL report of 30 September 2020 showed that out of a total of 54,128 complaints about COVID-19, 70.3% (around, 38,052 cases) concerned the “Health and Social Assistance” sector. The subanalysis of the data showed that the most affected professionals were “health technicians” (nurses, midwives, podiatrists, physiotherapists, speech therapists, orthopedists–ophthalmology assistants, neuro- and psychomotor therapists of developmental age, psychiatric rehabilitation technicians, professional educators, occupational therapists) with 39.2% of the total cases followed by qualified professions in health and social services (social health workers) (20%), doctors (10.1%), and unskilled personnel (auxiliaries, stretcher-bearers) (4.7%). Among the remaining categories, social assistance operators (careers) stand out, accounting for 8.9% of cases.

The data set showed a peculiar temporal and geographical trend (15, 16). Most of the positive cases and deaths for all sectors occurred between February and May. Similarly, the cases of COVID mainly affected the regions of the northwest (55.1%), followed by the northeast (24.4%), the center (11.9%), the south (6.2%), and from the islands (2.4%). The subanalysis carried out on the category of physicians showed slightly different data, with 67% of cases concerning the northern regions, 20% concerning the center, 9% the south, and 4% the islands. The positivity among the category of doctors concerning the global computation of INAIL complaints went from 10.3% (March–May) to 5.7% (June–September).

Another aspect investigated is that of mortality: in fact, the report describes 319 fatal cases due to COVID-19 (about one-third of the deaths reported since the beginning of the year and one incidence of 0.9% compared to the total of national deaths from COVID-19 communicated by the ISS as of September 30); of these, 35.7% died in March, 54.5% in April, 6.0% in May, 1.6% in June, 1.9% in July, 0.3% in August, and no cases reported in September.

The analysis by geographic origin shows a distribution of deaths of 56.7% in the northwest (Lombardy, 41.7%), 13.8% in the northeast (Emilia Romagna, 9.7%), by 11.6% in the center (Lazio, 4.7%), by 16.0% in the south (Campania, 7.2%), and of 1.9% in the islands (Sicily, 1.9%). The provinces with the most deaths are Bergamo (11.6%), Milan (8.2%), Brescia (7.8%), and Naples (6.0%). The analysis by profession of the injured person shows that about one-third of deaths concerns health and social assistance personnel. In detail, the more categories affected by the deaths are those of health technicians (58% are nurses), with 9.5% of codified cases and doctors with 6.9%, followed by socio-health workers with 5.1%, non-qualified personnel in health services (auxiliary, porters, stretcher-bearers) with 3.6%, and social welfare workers with 3.3%, and finally the specialists in the life sciences (toxicologists and pharmacologists) with 2.2%.

A very recent study considered the number of deaths from COVID-19 on the entire population of HCWs in 37 countries. The number of deaths in Italy was 0.35 per 100,000, second only to Mexico (0.9/100,000) and Azerbaijan (0.44/100,000) (17, 18). At present, it has not yet been investigated why the ratio of deaths to total workers regarding Italy is among the highest in the world (17).

However, the variables for explaining this difference can be divided into two main categories: (1) those that occurred when exposure to the SARS-CoV-2 among healthcare professionals had not yet been described and (2) those that emerged after the state of emergency became clear (18).

About physicians, in a first pre-emergency phase (during which there was a total unawareness of the importance of COVID-19 outbreak on public health), some medical fields were more penalized than others (e.g., those with a high number of contacts or those requiring the execution of procedures involving the formation of aerosols).

According to EUROSTAT statistical data, it appears that Italian doctors hold the European record with regards to age, with an average age of 55 years. A further reflection is possible if we compare the data with states such as Germany (GE) or Austria (AT), in which the average age of the population is equal or higher than the Italian population. According to the report, in Italy, the percentage of those over 65 years old was 18.1%, between 55 and 64 years old was 37.7%, while that of over 35 years old was 8.6%. In Germany and Austria, the over 65-year-olds accounted for 6.4 and 6.1%, those in the 55–64-year-old age group 38.5 and 25.4%, while the under 35-year-olds for 20.7 and 18.7%, respectively. The health policies of the last decade, characterized by the lengthening of the retirement age and the hiring freeze, have resulted in the average age of doctors in the national health service moving from 50.8 years in 2010 to 52.9 years in 2017 (19).

At the onset of the epidemic, the disease's high transmissibility was underestimated, and therefore, the use of suitable PPE was not strongly recommended. Simultaneously, due to the lack of knowledge on transmission routes, the need for specific recommendations made it necessary to apply guidelines for previous coronaviruses, such as the Middle East respiratory syndrome coronavirus (MERS-CoV) and SARS-CoV-2, which have different characteristics (20, 21).

Although these indications have proven to be useful for COVID-19, measures should be updated in accordance with recent data. Indeed, unlike other coronaviruses, SARS-CoV-2 can be transmitted from asymptomatic patients (22).

Another problem was the initial lack of knowledge of the transmission routes of SARS-CoV-2. SARS-CoV-2 can probably remain in an aerosol suspension for up to 16 h (23). Moreover, fecal–oral (24) and ocular (25) routes might also be crucial in limiting the diffusion of the ongoing pandemic even though they are not fully understood. In the next phase, when the state of emergency became evident, the numerous previously observed variables were combined with others, such as the initially limited availability of PPE, the low rate of staff turnover (due to the shortage of collaborators), and the failure to adapt medical liability to the moment of emergency to facilitate the use of emerging therapies (26, 27).

### COVID-19 and HCWs in Italy: The FNOMCeO Report: Differences Between Public and Private Physicians

**Figure 2:**
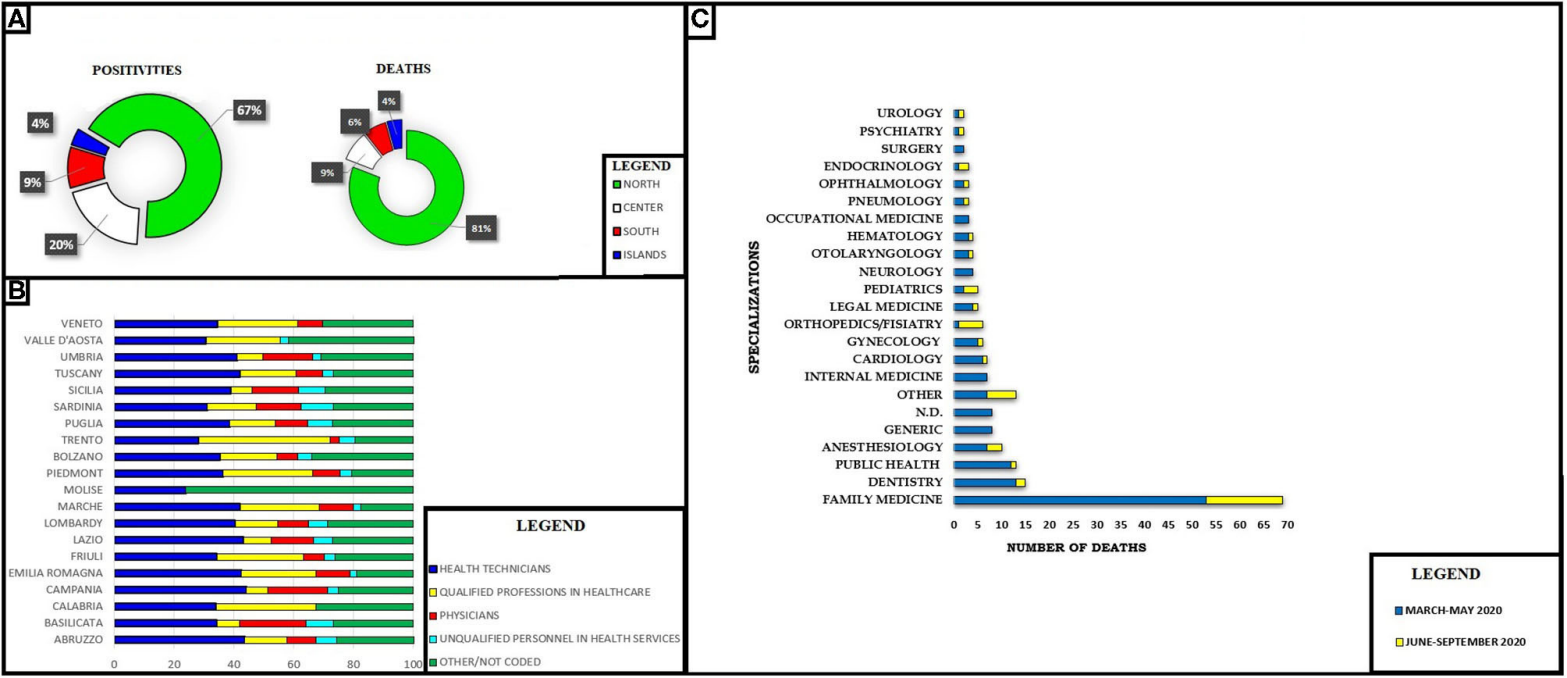
**(A)** Coronavirus disease 2019 (COVID-19) and Italian physician's positivities and deaths. North: Valle d' Aosta, Emilia Romagna, Friuli Venezia Giulia, Liguria, Lombardy, Piedmont, Veneto, Trento, Bolzano. Center: Abruzzo, Lazio, Marche. South: Puglia, Basilicata, Calabria, Campania, Tuscany, Umbria Islands: Sardinia, Sicily. **(B)** COVID-19 positivities and occupational sector in Italian regions. **(C)** Number of deaths for specialization. N.D., not declared; Other, thermal medicine, radiology, penitentiary medicine, homeopathy, geriatrics, bioethics.

A recent document from the Federazione Nazionale degli Ordini dei Medici Chirurghi e degli Odontoiatri (FNOMCeO), the national federation of Italian medical doctors and dentists, provided data on the deaths during the epidemic with data on the specialization of each deceased physician (13, 28). These data are only apparently in contradiction with those of INAIL previously provided; for many Italian doctors (for example, general practitioners, freelancers), they are not protected by this Institute or continue to work privately after retiring as public employees (Figure 2).

The most affected active categories were those of general practitioners (GPs) and dental practitioners (DPs). Regarding GPs, it is possible to postulate that these figures are due to the high number of accesses. Especially in February–March, the ordinary PPE supplies were insufficient to deal with SARS-CoV-2.

Specifically, for GPs, the scarce use (due to shortages of supplies) of individual protection devices and intensive exposure to biological hazards might have played a role. In the case of dentists, the production of aerosols during the procedures carried out, and the lack of interventions for environmental sanitation between one intervention and another, could have played a role.

## Discussion and Future Challenges

Although the situation has improved at present, the near future presents several challenges for Italian HCWs. The first challenge is returning to pre-emergency activities; even though the number of infections has decreased, it has not yet reached zero. A return to activities as before could lead to a spread of the virus. For this reason, it is essential to strive for greater knowledge of the virus that would allow the application of adequate preventive and sanitization techniques on which there is still no shared strategy. However, there have been interesting and valid proposals (29).

A second critical point is represented by the possibility of pharmacologically preventing the disease, especially in the event of a new epidemic wave. Unfortunately, due to the decrease in the number of infected people on which to test a vaccine, an effective vaccine has not yet been put in place nor has it been ascertained that immunization is possible. Even the trials on chemoprophylaxis and the results of the application of this strategy were not encouraging. However, it is necessary to consider that HCWs exposed to high biological risks can represent this virus's source (30–32). One of the most topical issues concerns the use of vaccines produced up to now. From August to today, there have been announcements on the discoveries of various vaccines, some proteins (Gam-COVID-Vac), and others for the first time in the history of “genetic” type to mRNA (MRNA-1273 and BNT162b). Especially for the latter category, no long-term safety data are currently available. This point raises ethical and moral questions, especially if we consider that this category of vaccine is being used for the first time and that HCWs—as a high-risk category and potential source of the outbreak—could be required to have compulsory vaccination for access to work (33–35).

Until effective prophylactic protocols are elaborated, the continuous adaptation of the guidelines based on the knowledge of the virus's characteristics is essential to minimize the biological risks (36–38).

The spread of the pandemic has given rise to important ethical and medicolegal dilemmas (38). In fact, in the first phase of the pandemic, due to the high biological risk, no or limited autopsies were carried out. This has contributed to slowing down the accumulation of knowledge on the effects of the disease and the therapeutic management of patients. Knowledge of the pathogenesis and its consequences will also be important to evaluate any permanent damage reported by the HCWs in carrying out their work.

Another aspect concerns informed consent and visits. In current clinical practice, consent is extended to each patient who accesses a visit with questions about possible contacts and symptoms attributable to COVID-19. Another aspect concerns the development of telemedicine for which remote evaluations have been developed, the carrying out of which was unthinkable until 2019 (39).

A last but very important problem for Italian HCWs is a professional responsibility. Until July 2020, Italy remained one of the few countries in the world not to have provided a criminal shield for those who provided healthcare during the epidemic, especially in the first period (40–42).

Class action suits against doctors, healthcare facilities, and Italian HCWs have been taken and advertised, and this battle is on two fronts: (1) the one against SARS-CoV-2 not yet finished and (2) the one in court that will probably start soon. In particular, in the current medicolegal practice, requests for evaluations are frequent, not so much for fatal cases linked to COVID-19 but rather for delays and omissions due to the “state of emergency.”

## Conclusion

The battle between the Italian Healthcare Workers and COVID-19 has been characterized by highly criticality moments and has resulted in a high number of infections and deaths. The emergency, which underlines the fragility of a state-of-the-art health system, such as the Italian system, cannot be considered complete despite the great progress in the number of infected people, intensive care patients, and deaths. Among the critical points highlighted are the need to acquire further knowledge about the virus, of validating shared sanitation techniques for the resumption of daily health activities, and of developing prevention techniques.

An Italian peculiarity is represented by the need to approve a penal shield, which is also present in other countries and would allow HCWs to work with peace and security regarding medical liability, even in times of crisis.

## Data Availability Statement

Publicly available datasets were analyzed in this study. This data can be found here: https://portale.fnomceo.it/elenco-dei-medici-caduti-nel-corso-dellepidemia-di-covid-19/; https://www.inail.it/cs/internet/docs/alg-scheda-tecnica-contagi-covid-30-settembre-2020.pdf.

## Author Contributions

MN, Ed'A, and PN conceived of the presented idea. MF and JL developed the theory. MN wrote the manuscript in consultation with Ed'A, PN, JL, and MF. All authors contributed to the article and approved the submitted version.

## Conflict of Interest

The authors declare that the research was conducted in the absence of any commercial or financial relationships that could be construed as a potential conflict of interest.
